# Combination therapy with omalizumab and an immune-suppressive agent for resistant chronic spontaneous rrticaria - A real-life experience^☆^

**DOI:** 10.1016/j.waojou.2020.100448

**Published:** 2020-08-04

**Authors:** Ramit Maoz-Segal, Tanya Levy, Soad Haj-Yahia, Irena Offengenden, Mona Iancovich-Kidon, Nancy Agmon-Levin

**Affiliations:** a Clinical Immunology, Angioedema and Allergy Unit, Center for Autoimmune Diseases, Sheba Medical Center, Tel Hashomer, Israel; b Sackler School of Medicine, Tel Aviv University, Ramat-Aviv, Israel

**Keywords:** Resistant CSU, Omalizumab, Cyclosporine, CSU phenotypes, Autoimmunity, Autoallergy, CSU, chronic spontaneous urticaria, r-CSU, resistant chronic spontaneous urticarial, Or-CSU, Omalizumab responsive CSU, IgE, immunoglobulin E, FceR1, high-affinity receptor for Immunoglobulin E, IL-24, interleukin 24, ANA, anti-nuclear antibodies

## Abstract

**Background:**

Chronic Spontaneous Urticaria (CSU) is a relatively common immune mediated disease that can be effectively treated nowadays. Nevertheless, for some patients remission cannot be achieved following current treatment recommendations, defined as resistant CSU (r-CSU). Treating r-CSU is challenging, and, currently, there are no recommended interventions. In this real-life study we describe successful therapy of 18 r-CSU patients using an "intensified protocol" of anti-IgE-antibody (omalizumab) concomitantly with an immunosuppressant. We defined the r-CSU phenotype and compared it to omalizumab-responsive CSU (Or-CSU) phenotype.

**Methods:**

Clinical and serological data of 72 CSU patients (ie, 18 r-CSU and 54 age and sex matched Or-CSU) were retrospectively collected and analyzed. All patients were diagnosed with CSU for ≥6 months and treated at the Sheba Medical Center during 2013–2018.

**Results:**

Of 289 CSU patients, 18 (6%) were diagnosed with r-CSU and treated with the "intensified protocol" including omalizumab and cyclosporine-A (16p), methotrexate (1p), and azathioprine (1p). Of which, 14/18 (78%) achieved complete remission, 2/18 (11%) partial remission, and 2/18 (11%) no remission. During follow-up no serious adverse events were documented. r-CSU patients received higher doses of antihistamine (p < 0.0001) and omalizumab (425 ± 58 mg/month vs. 283 ± 86 mg/month; p < 0.0001) compared to Or-CSU. The r-CSU phenotype was linked with concomitant autoimmunity (p = 0.0005) and a lower level of IgE prior to initiation of therapy (p = 0.027).

**Conclusion:**

r-CSU may be a distinct CSU phenotype characterized by severe disease, concomitant autoimmunity, and lower baseline-IgE levels (low "autoallergy"). An "intensified protocol" with omalizumab and an immunosuppressive agent was found to be efficacious and safe for r-CSU. Further larger studies are required to verify these results.

## Introduction

Chronic Spontaneous Urticaria (CSU) is an immune mediated disorder that affects up to 1% of the general population, and it is characterized by hives, pruritus, and frequent angioedema.^1^ This chronic condition is associated with various co-morbidities, decreased quality of life, and reduced ability to maintain normal activities.^2^, ^3^, ^4^ Two immune mechanisms were linked with CSU pathogenesis. “Type I″ response that is mediated by IgE antibodies, but unlike classic allergy, these IgE antibodies are directed at auto-antigens (e.g. IL-24).^5^ This mechanism was recently termed "Auto-allergy". “Type II” immune responses are autoimmune in nature and are mediated by IgG autoantibodies directed against the high-affinity receptor for IgE (FceR1) or membrane-bound IgE.^6^^,^^7^

Regardless of the dominant immune-mechanisms, a strong link was observed between CSU and concomitant autoimmunity, namely, systemic or organ specific autoimmune diseases and/or autoantibodies. A myriad of autoimmune diseases such as pernicious anemia, psoriasis, vitiligo, type I-diabetes mellitus, Celiac disease, Graves' disease, and Hashimoto's thyroiditis, are more prevalent among CSU patients.^8^^,^^9^ Equally, systemic autoantibodies such as anti-Thyroperoxidase (Anti-TPO) and anti-nuclear antibodies (ANA) were documented to be more common in CSU. Like other autoimmune diseases, CSU was linked with female gender and family history of autoimmunity.^9^, ^10^, ^11^

Treatment guidelines for CSU were published by the EAACI/GA2LEN/EDF/WAO and revised in 2018.^12^^,^^13^ Similar guidelines were published in 2014 by the American group representing the Joint Task Force on Practice Parameters (JTFPP),^14^ the Israeli Association for Allergy and Clinical Immunology (IAACI), and other groups.^15^^,^^16^ In all algorithms for CSU treatment, a stepwise approach is recommended. The first step includes regular doses of second generation antihistamines followed by up to four-fold incremental increase in antihistamine dose. Leukotriene antagonists were included in former guidelines (ie, 2013) as part of the third line of therapy for CSU. However, in 2018 these drugs were no-longer advised as there is scarce evidence for their efficacy. Thus, currently only omalizumab and cyclosporine are included as third and fourth line of therapy, respectively. Of note, in Israel the Ministry of Health instructions, issued in 2015, require leukotriene antagonist therapy prior to omalizumab. This algorithm revolutionized CSU management in the last decade and set the goal of achieving complete remission of symptoms in all patients. However, presently in up to 15% of patients disease remission can not be achieved. This non-responding variant of disease is defined resistant CSU (r-CSU).^17^

Treatment of r-CSU in a clinical setting continues to be a challenge. Development of new potent drugs is 1 way to overcome r-CSU, whereas combination of available modalities may be another path to achieve disease remission. In the current study we describe 18 r-CSU patients that were successfully treated with a combination of omalizumab and an immunosuppressive medication (ie, intensified protocol). Additionally, we compared r-CSU patients with CSU patients that responded favorably to omalizumab (Or-CSU) in order to define the r-CSU phenotype. We believe the knowledge obtained from this real-life experience of treating r-CSU may help physicians worldwide.

## Methods

In this single center observational study, we retrospectively evaluated 72 Israeli CSU patients treated during January 2013 to December 2018. In this period 289 CSU patients were treated in the Clinical Immunology and Allergy Department, at Sheba Medical Center, Israel. Eighteen^18^ of these patients were diagnosed with r-CSU, and 54 consecutive age and gender matched CSU patients that were omalizumab responsive (Or-CSU) served as a control group. r-CSU was defined following failure to achieve disease remission utilizing all steps of the recommended protocols (ie, high dose second generation antihistamines, 0malizumab, immunosuppressant). Notably, although Urticaria Activity Score (UAS) was not performed regularly in this study, very low disease activity (eg, few lesions a week and mild intermittent itch, which may be comparable to a low UAS score of <6), was regarded as remission. Demographic disease features (eg, disease duration, co-presence of inducible urticaria, angioedema, etc) comorbidities, laboratory parameters (as CRP and ESR; autoantibodies, baseline-IgE levels etc), as well as treatment doses and outcomes were collected and analyzed. Patients were followed every 4 weeks. Blood analysis was preformed prior to initiation of omalizumab and/or immunosuppressive therapy and thereafter, periodically, as commonly practiced (ie, complete blood count, transaminase levels, creatinine level, and cyclosporine levels, if relevant). This study received approval by the institutional ethics committee and fulfilled the ethical guidelines of the declaration of Helsinki (Edinburgh 2000).

In the study most patients received omalizumab 300 mg/month (the recommended dose by the Israeli Ministry of Health) and fewer 150 mg/month, if was sufficient for complete response. When a partial response to omalizumab was observed, dose escalation was attempted (eg, 450 mg/month) if approved by the patient's health maintenance organization (HMO). Notably, all patients received at least 2 courses of oral glucocorticoids and ≥8 weeks of montelukast within the 12 months prior to initiation of omalizumab (as required by the Israeli regulation). Immunosuppressive drugs were given at the following doses: Cyclosporine 1–3 mg/kg/day, azathioprine 1–2.5 mg/kg/day, and methotrexate up to 20 mg/week. Doses were tailored individually and were raised gradually according to disease severity and drug tolerability. Response to therapy was determined after 3–6 months of initiation of immunosuppressant or omalizumab respectively. Treatment outcomes were defined as *complete remission* if full withdrawal of glucocorticoids therapy was achieved and disease activity was improved by ≥ 80% (eg, .hives, rash, angioedema) compared to baseline, in other words if disease activity was very low and/or non-active. *Partial response* was withdrawal of glucocorticoids therapy and decrease of symptoms by 50–80% and *lack of response* was if improvement criteria were not met (eg, less than 50%), as reported by the patient and physician. Serious adverse events were determined as those that required hospitalization or stopping of an immunosuppressive drug. CSU was considered to be “associated with autoimmunity” if concomitant overt autoimmune disease and/or high titers of autoantibodies were present.

### Statistics

Statistical analysis was performed using SPSS 24.0. For all tests p < 0.05 was considered statistically significant. Continuous variables were described as mean ± SD, and categorical variables as percentages. Comparisons between cases (r-CSU group) and controls (Or-CSU group) were analyzed by Chi-square test or Fisher's exact test as appropriate for categorical variables, and by Student's T-test or Mann-Whitney for continuous variables.

## Results

**Fig. 1:**
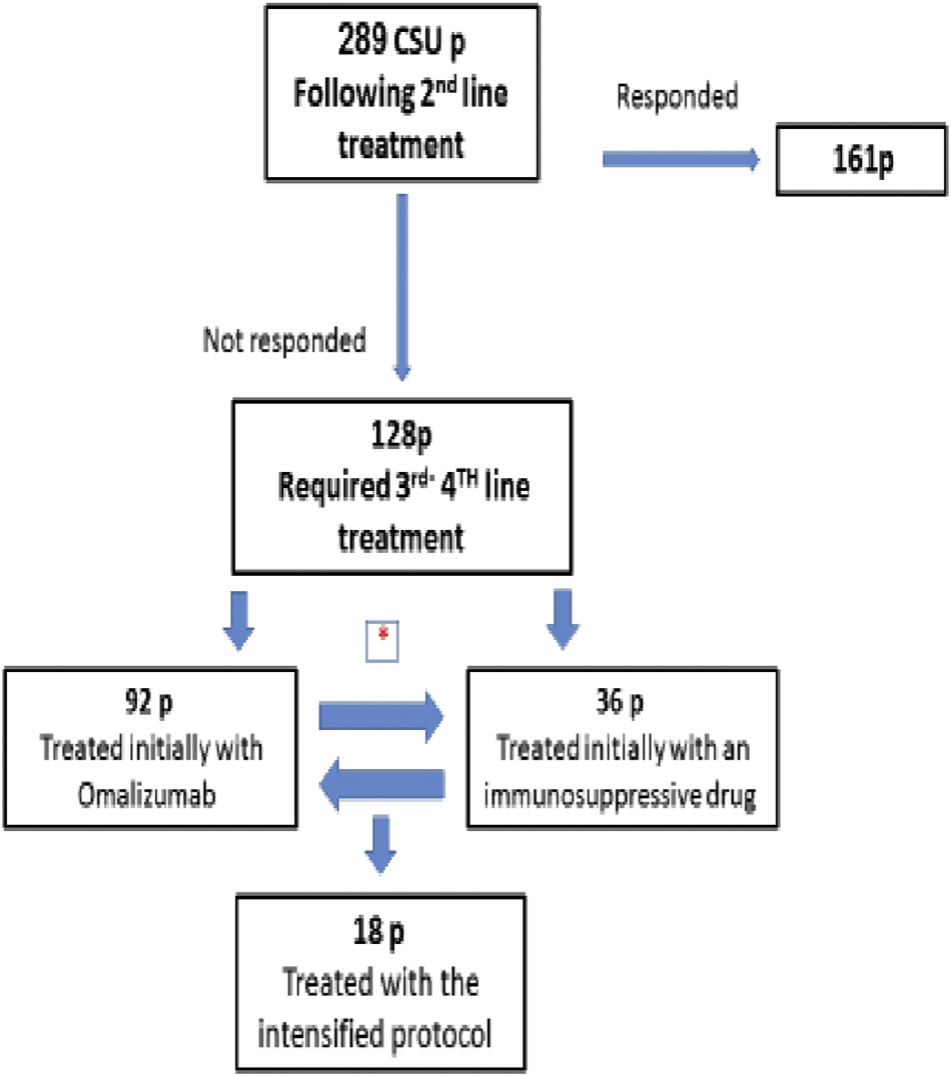
2nd line –high dose anti-histamine, **3**rd**-4**th **lines**- Omalizumab or immunosuppressant; **∗** Cross over to the alternative drug if remission was not achieved; **Intensified protocol** - Omalizumab and immunosuppressant

In our cohort of 289 CSU patients, 161 (56%) achieved remission while treated with regular or high dose antihistamines, and 128 (44%) required third/fourth lines of treatment. Of these patients, 92/128 (72%) were initially treated with omalizumab, and 36/128 (28%) initially treated with immunosuppressive drug. If a response was not achieved patients were diagnosed with r-CSU, as both drugs (either omalizumab or cyclosporine) did not induce remission (Fig. 1). Subsequently, r-CSU patients were treated with the “intensified protocol” that includes omalizumab at the highest dose (300 mg/month [3p] and 450 mg/month [15p]) in conjunction with an immunosuppressive drug (Cyclosporine [16p], Methotrexate [1p] or Azathioprine [1p]). In the current study the mean duration of treatment with the “intensified protocol” was 14 ± 8 months (range 9–40 months), whereas the whole follow-up period was 57 months. The responses to this “intensified protocol” were complete remission in 14/18 (78%; of which 1 was treated with methotrexate), partial response in 2/18 (11%; of which 1 was treated with azathioprine), and no response in 2/18 (11%) of patients. Noteworthy, during follow-up, 6/14 (43%) patients that achieved complete remission could decrease therapy: 4/6 (67%) reduced 1 of the drugs, and 2/6 (33%) patients discontinued both drugs with no relapse. No serious adverse events were documented during follow up. Dose modifications of cyclosporine were required in 2 patients due to high blood pressure and mild elevation of creatinine level (1.5 mg/dl) for another; both normalized thereafter.

### r-CSU compared to omalizumab responsive disease (Or-CSU)

Our 18 r-CSU patients were age and gender matched with 54 consecutive CSU patients that achieved complete remission with omalizumab (Or-CSU). As can be expected r-CSU patients received higher doses of antihistamines, omalizumab and montelukast compared to Or-CSU group (Table 1). Associated autoimmunity was more common among r-CSU group vs Or-CSU (55% *vs* 20%, p = 0.0005). In contrast, higher levels of IgE prior to initiation of omalizumab therapy were less prevalent among r-CSU patients compared to Or-CSU (18% vs 41%, respectively; p = 0.027) although baseline IgE levels were available only for 11/18 r-CSU and 29/54 Or-CSU patients compared.

**Table 1:** Demographics and clinical manifestations of r-CSU vs. Or-CSU groups.

	r-CSU group (n = 18)	Or-CSU group (n = 54)	P value
Mean age (years; mean ± SD)	45 ± 16	46 ± 18	NS
Male gender	2 (11%)	15 (28%)	NS
Disease duration (years; mean ± SD)	5 ± 5	5 ± 6	NS
Concomitant Angioedema	14 (77%)	43 (79%)	NS
Concomitant inducible urticaria	5 (27%)	22 (40%)	NS
High IgE levels (above upper limits) prior to therapy	2/11 (18%)	12/29 (41%)	p = 0.027
Concomitant Autoimmunity	10 (55%)	11 (20%)	p = 0.0005
Treated with x4 fold anti H1	18 (100%)	15 (46%)	p < 0.0001
Treated with Montelukast	18 (100%)	30 (55%)	p < 0.0001
Omalizumab dose (mg/month)	425 (±58)	283 (±86)	p < 0.0001

**NS**- Non-significant; **Montelukast-**leukotriene antagonist; **Omalizumab** – anti-IgE monoclonal antibody

## Discussion

CSU therapy changed dramatically in the last decade and complete remission of symptoms was set as an achievable goal. Nevertheless, a sizable proportion of patients suffer from resistant disease (r-CSU), despite improvements in therapy, for which recommended interventions are lacking. Multiple new medications are being investigated for this resistant phenotype of CSU: 2 novel anti-IgE antibodies (ligelizumab and UB-221), the anti-cytokine monoclonal antibodies (dupilumab, reslizumab, mepolizumab, and benralizumab), a CRTh2 antagonist, a monoclonal antibody to Siglec-8 (AK002), Bruton's tyrosine kinase inhibitors (fenebrutinib and Lou064), and a Syk inhibitor, as recently reviewed by Maurer et al.^18^ These are intriguing therapeutic modalities that are still in different stages of research, and might be found efficacious for r-CSU, but are expected to be of high cost. This leaves many patients worldwide without adequate treatment. In this real-life clinical study we report for the first time, to the best of our knowledge, results of combining 2 modalities that are currently available and reimbursed in most countries for the treatment of r-CSU, namely the approved anti-IgE monoclonal antibody, omalizumab,^19^ with an immunosuppressant agent. This “intensified protocol” was efficacious in 16/18 of r-CSU patients, of which 6 patients were able to withdrawal 1 or both drugs and maintain remission during follow up.

In the present study, in the context of real-life clinical practice, r-CSU was diagnosed in 6% of our entire CSU cohort, and in 14% of those that required the third-fourth lines of therapy. This is similar to previous reports. For instance, in a former study r-CSU was reported in 7% of the general CSU population,^20^ and lack of response to omalizumab in 14–33% of treated patients. In a multicenter retrospective analysis from Israel, CSU remission was achieved in 86% of patients treated with omalizumab. In other studies from Latin America and Australia, 80% and 67% of CSU patients, respectively, responded favorably to this intervention.^17^^,^^20^, ^21^, ^22^, ^23^, ^24^, ^25^ In contrast, a lower success rate of omalizumab was recently documented in a worldwide multicenter real-life study, the AWARE study. The relatively lower success of omalizumab was attributed to lack of adherence to the recommended guidelines.^26^^,^^27^ Furthermore, recent publications report that urticaria-guideline recommendations, which contribute to a higher quality of patient care, are not followed precisely by 20% of physicians.^28^ An additional explanation for this relatively high non-response rate may be the lack of up-dosing omalizumab that was utilized in our study.

The most commonly used immunosuppressant in our “intensified protocol” was cyclosporine. The use of cyclosporine is supported by data from several clinical studies^29^ and most guidelines. Admittedly, its safety profile is lower compared to omalizumab with potential side effects such as elevated creatinine, hypertension, fatigue, gastrointestinal problems, gingivitis, and headaches.^30^ Yet, up to 2015 cyclosporine was used prior to omalizumab in many countries, as well as in Israel. In 2018 guidelines it is recommended to use cyclosporine as a alternative treatment option for omalizumab non-responders.^31^ In a recently published small meta-analysis of cyclosporine efficacy and safety in CSU, the results support its effectiveness, and it is suggested that its safety is dose dependent.^32^ Following these recommendations, the “intensified protocol” includes low doses of cyclosporine (1–2 mg/kg) concomitantly with regular or high doses of omalizumab. No significant adverse events were observed, although close monitoring and dose adjusment of cyclosporine was required for some patients. Our results support the recently published recommendations to consider adding low dose cyclosporine to omalizumab in r-CSU patients.^33^ Data regarding methotrexate and azathioprine for CSU is scarce.^31^^,^^34^^,^^35^ Although both are used as “steroid sparing” drugs for many autoimmune conditions, they are seldom recommended for CSU. In the current study, as described, only 2 patients that could not receive cyclosporine received these drugs.

We compared the 2 phenotypes of CSU in our cohort, namely r-CSU vs. Or-CSU, trying to find biomarkers or other clinical parameters defining each group. Indeed, recent reports characterized 2 possible endotypes according to the type of mast cell degranulation signals: type I autoimmune CSU mediated with IgE autoantibodies to auto allergens (or *autoallergy*), and type IIb autoimmune CSU mediated with autoantibodies that target activating mast cells receptors. These 2 endotypes of autoimmune hypersensitivity have been postulated as being etiologic in most CSU patients. There is a need for development of diagnostic tests to differentiate these 2 endotypes of CSU in order to tailor the appropriate treatment and help each patient achieve remission.^36^ Intriguingly, we found 2 major differences between r-CSU and Or-CSU in our study: High total IgE levels (associated with type I autoimmunity) were related to Or-CSU phenotype (18% vs 41%, respectively) while concomitant autoimmunity (which may support type II autoimmunity) was related to r-CSU phenotype (55% *vs.* 20%, respectively).

The predictive role of specific and total IgE level in CSU was evaluated in recent studies, and a particular link with response to omalizumab has been suggested.^17^^,^^36^^,^^37^ Additionally, more than 200 IgE autoantigens were detected in CSU patients, but not in healthy subjects. Some of these autoantibodies, such as IgE anti-IL-24 antibodies, are possible biomarkers, and their levels correlate with disease severity.^5^^,^^38^, ^39^, ^40^ On the other hand, low total IgE level prior to initiation of therapy was associated with a slower response to omalizumab, reduced efficacy of this drug and a shorter time to disease relapse after omalizumab discontinuation.^41^, ^42^, ^43^ Similarly, we have found in our cohort, that lower baseline IgE correlated with r-CSU, further supporting baseline IgE levels as a marker of response to anti-IgE therapy. Our results are compatible with those demonstrated in the PURIST study which demonstrated more severe disease and markedly lower IgE levels among patients diagnosed with autoimmune CSU compare to non-autoimmune CSU patients.^44^ Notably, better understanding of CSU endotypes is needed, not only for choosing the better treatment for each patient, but also, with no less importance, for evaluation of treatment duration following remission. For example, in a recent study accepted for publication, it was confirmed that it is possible to half omalizumab dose and maintain efficacy in a large subgroup of CSU patients, which had an excellent response to full dose omalizumab. Unfortunately, none of the currently available biomarkers of efficacy or severity of disease, including IgE level, were able to predict which patients will relapse following omalizumab dose reduction.^45^ Our results stand in agreement with findings from former studies in which associated autoimmunity was linked with difficult to treat CSU. A higher rate of r-CSU was documented among anti-nuclear antibodies (ANA) positive patients, compared to those with undetectable ANA.^10^ Moreover, in several studies the autologous serum skin test, which supports the presence of IgG autoantibodies directed against FcεRI IgE,^46^^,^^47^ was associated with a slower response to therapy particularly to omalizumab.^48^ Thus, it seems that the prevalence of autoantibodies and/or autoimmune comorbidities, as suggested by the results from our study, may be considered a marker of resistant/severe CSU.

Our study has several limitations derived from its retrospective, observational nature. Nonetheless, these real-life data presented from our tertiary center stand in agreement with data from another large Israeli multicenter study, as well as from studies from other countries. All these studies highlight the unmet need in treating r-CSU, as well as the need for an individual tailored approach to treating these patients according to disease endotypes. Additionally, we did not measure routinely UAS, nor IgG-anti-IgE receptor antibodies which are of great importance in the pathogenesis of CSU.^49^ These parameters are not used in common practice in Israel and these specific antibodies are usually measured in research laboratories and cannot be measured in our country. Further prospective studies of r-CSU, which will examine more specific markers, are required.

## Conclusions

In this clinical observational retrospective study, an “intensified protocol" using omalizumab and an immunosuppressant concomitantly was found to be efficacious and safe for treating r-CSU patients, and thus may be an alternative solution for this difficult condition. In addition, r-CSU patients were found to have more concomitant autoimmunity and lower baseline-IgE levels and, as such, may be regarded as a distinct phenotype of CSU, characterized by severe disease. Further prospective controlled larger studies are required to verify this approach.

## Author contribution

**Dr. Ramit Maoz-Segal, Mrs. Tanya Levi, Prof. Nancy Agmon-Levin**: Contributed to the study conception and design. Contributed to acquisition of data, analysis and interpretation of data. Drafted the article. Given a final approval of this version to be published and agrees to be accountable for all aspects of the work related to its accuracy and integrity.

**Mrs. Tanya Levi**: She is a medical student and has no academic degree. Contributed to the study conception. Contributed to analysis and interpretation of data. Drafted the article. Given a final approval of this version to be published and agrees to be accountable for all aspects of the work related to its accuracy and integrity.

**Dr. Soad Haj-Yahia, Dr. Irena Offenganden, Dr. Mona Iancovich-Kidon:** Contributed to interpretation of data. Reviewed the article including the references. Given final approval of this version to be published and agrees to be accountable for all aspects of the work related to its accuracy and integrity.

This manuscript has been read and approved for publication by all authors.

All data and material are available by demand.

## Funding source

Not applicable.

## Declaration of Competing Interest

The authors have nothing to declare.
